# 5-Bromo-3-(3-fluoro­phenyl­sulfin­yl)-2-methyl-1-benzofuran

**DOI:** 10.1107/S1600536812032394

**Published:** 2012-07-18

**Authors:** Hong Dae Choi, Pil Ja Seo, Uk Lee

**Affiliations:** a Department of Chemistry, Dongeui University, San 24 Kaya-dong, Busanjin-gu, Busan 614-714, Republic of Korea; b Department of Chemistry, Pukyong National University, 599-1 Daeyeon 3-dong, Nam-gu, Busan 608-737, Republic of Korea

## Abstract

In the title compound, C_15_H_10_BrFO_2_S, the 3-fluoro­phenyl ring makes a dihedral angle of 85.0 (1)° with the mean plane [r.m.s. deviation = 0.008 (2) Å] of the benzofuran fragment. In the crystal, mol­ecules are linked by weak C—H⋯O hydrogen bonds and a Br⋯O contact [3.200 (3) Å]. The crystal structure also exhibits slipped π–π inter­actions between the benzene and furan rings of neighbouring mol­ecules [centroid–centroid distance = 3.619 (4) Å and slippage of 1.389 (4) Å]. In the 3-fluoro­phenyl ring, the F atom is disordered over two positions with site-occupancy factors of 0.583 (5) and 0.417 (5).

## Related literature

For background information and the crystal structures of related compounds, see: Choi *et al.* (2010*a* ▶,*b* ▶, 2012 ▶). For a review of halogen bonding, see: Politzer *et al.* (2007 ▶).
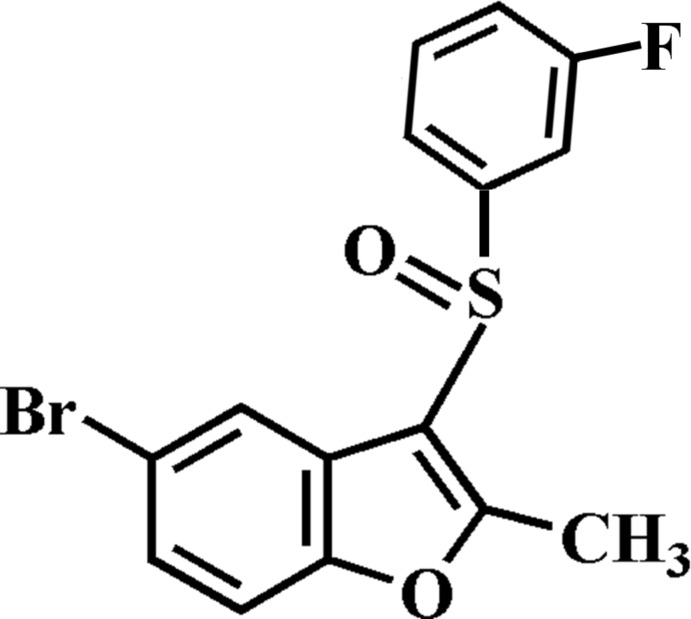


## Experimental

### 

#### Crystal data

C_15_H_10_BrFO_2_S
*M* *_r_* = 353.20
Monoclinic, 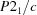

*a* = 13.0488 (4) Å
*b* = 11.1874 (3) Å
*c* = 9.9295 (3) Å
β = 105.709 (2)°
*V* = 1395.39 (7) Å^3^
*Z* = 4
Mo *K*α radiation
μ = 3.10 mm^−1^
*T* = 173 K
0.25 × 0.24 × 0.13 mm

#### Data collection

Bruker SMART APEXII CCD diffractometer
Absorption correction: multi-scan (*SADABS*; Bruker, 2009 ▶) *T*_min_ = 0.494, *T*_max_ = 0.746
13225 measured reflections
3460 independent reflections
2392 reflections with *I* > 2σ(*I*)
*R*_int_ = 0.048

#### Refinement

*R*[*F* ^2^ > 2σ(*F* ^2^)] = 0.043
*wR*(*F* ^2^) = 0.115
*S* = 1.03
3460 reflections
192 parameters
14 restraints
H-atom parameters constrained
Δρ_max_ = 0.61 e Å^−3^
Δρ_min_ = −1.10 e Å^−3^

### 

Data collection: *APEX2* (Bruker, 2009 ▶); cell refinement: *SAINT* (Bruker, 2009 ▶); data reduction: *SAINT*; program(s) used to solve structure: *SHELXS97* (Sheldrick, 2008 ▶); program(s) used to refine structure: *SHELXL97* (Sheldrick, 2008 ▶); molecular graphics: *ORTEP-3* (Farrugia, 1997 ▶) and *DIAMOND* (Brandenburg, 1998 ▶); software used to prepare material for publication: *SHELXL97*.

## Supplementary Material

Crystal structure: contains datablock(s) global, I. DOI: 10.1107/S1600536812032394/kp2435sup1.cif

Structure factors: contains datablock(s) I. DOI: 10.1107/S1600536812032394/kp2435Isup2.hkl

Supplementary material file. DOI: 10.1107/S1600536812032394/kp2435Isup3.cml

Additional supplementary materials:  crystallographic information; 3D view; checkCIF report

## Figures and Tables

**Table 1:** Hydrogen-bond geometry (Å, °)

*D*—H⋯*A*	*D*—H	H⋯*A*	*D*⋯*A*	*D*—H⋯*A*
C5—H5⋯O1^i^	0.95	2.50	3.429 (4)	167
C9—H9*A*⋯O2^ii^	0.98	2.44	3.269 (4)	142

Symmetry codes: (i) 
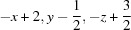
; (ii) 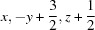.

**Fig. 1.**
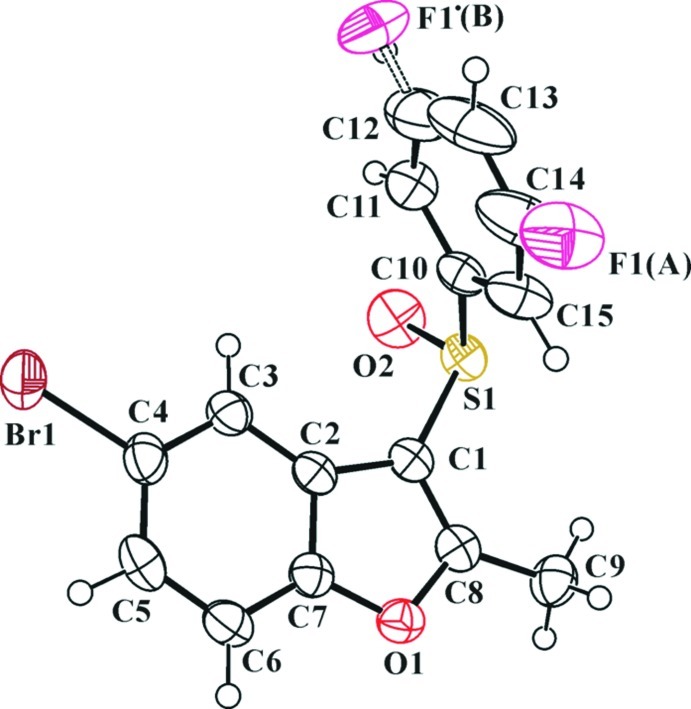
The molecular structure of the title compound with the atom numbering scheme. Displacement ellipsoids are drawn at the 50% probability level. H atoms are presented as small spheres of arbitrary radius. The F atom of the 3-fluorophenyl ring is disordered over two positions with site occupancy factors, from refinement of 0.583 (5)(part A) and 0.417 (5) (part B).

**Fig. 2.**
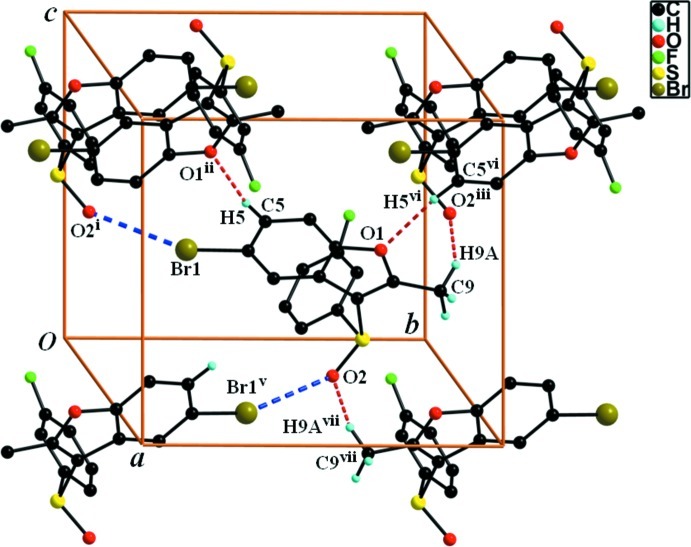
A view of the C—H···O and Br···O interactions (dotted lines) in the crystal structure of the title compound. H atoms non-participating in hydrogen-bonding and disodered part B atoms were omitted for clarity. [Symmetry codes: (i) x, - y + 1/2, z + 1/2; (ii) - x + 2, y - 1/2, - z + 3/2; (iii) x, - y + 3/2, z + 1/2; (v) x, - y + 1/2, z - 1/2; (vi) - x + 2, y + 1/2,- z + 3/2; (vii) x, - y + 3/2, z - 1/2.]

**Fig. 3.**
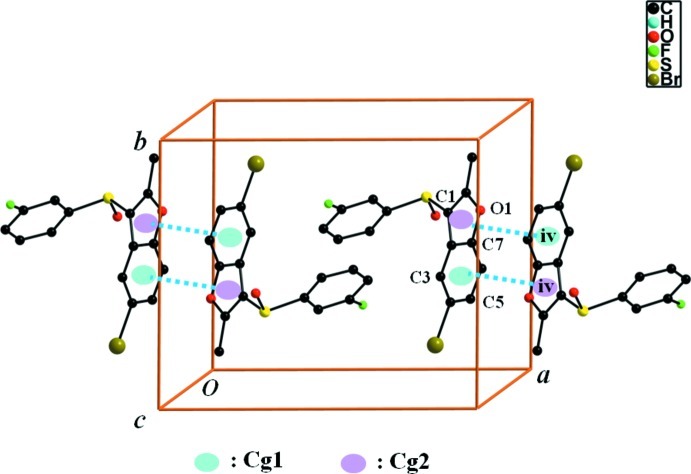
A view of the π–π interactions (dotted lines) in the crystal structure of the title compound. All H atoms and disordered part B atoms were omitted for clarity. [Symmetry codes: (iv) - x + 2, - y + 1, - z + 1.]

## supplementary crystallographic information

### Comment

As a part of our ongoing study of 5-bromo-2-methyl-1-benzofuran derivatives containing 3-(4-fluorophenylsulfinyl) (Choi *et al.*, 2010*a*), 3-(4-chlorophenylsulfinyl) (Choi *et al.*, 2010*b*), and 3-(4-methylphenylsulfinyl) (Choi *et al.*, 2012) substituents, we report herein the crystal structure of the title compound.

In the title molecule (Fig. 1), the benzofuran unit is planar with a mean deviation of 0.008 (2) Å from the least-squares plane defined by the nine constituent atoms. In the 3-fluorophenyl ring, the F atom is disordered over two positions with site-occupancy factors, from refinement of 0.583 (5)(part A) and 0.417 (5)(part B). The dihedral angle between the 3-fluorophenyl ring and the mean plane of the benzofuran ring is 85.0 (1)°. In the crystal structure (Fig. 2), molecules are connected by weak C–H···O hydrogen bonds (Table 1), and a Br···O interaction between the bromine and the oxygen of the S═O unit [Br···O2^i^ = 3.200 (3) Å, C4—BrI···O2^i^ = 159.51 (11)°] (Politzer *et al.*, 2007). The crystal packing (Fig. 3) also exhibits slipped π–π interactions between the benzene and furan rings of neighbouring molecules, with a Cg1···Cg2^iv^ distance of 3.619 (4) Å and an interplanar distance of 3.342 (4) Å resulting in a slippage of 1.389 (4) Å (Cg1 and Cg2 are the centroids of the C2-C7 benzene ring and the C1/C2/C7/O1/C8 furan ring, respectively).

### Experimental

3-Chloroperoxybenzoic acid (77%, 224 mg, 1.0 mmol) was added in small portions to a stirred solution of 5-bromo-3-(3-fluorophenylsulfanyl)-2-methyl-1-benzofurann (303 mg, 0.9 mmol) in dichloromethane (30 mL) at 273 K. After being stirred at room temperature for 4 h, the mixture was washed with saturated sodium bicarbonate solution and the organic layer was separated, dried over magnesium sulfate, filtered and concentrated at reduced pressure. The residue was purified by column chromatography (hexane–ethyl acetate, 2:1 v/v) to afford the title compound as a colourless solid [yield 74%, m.p. 393–394 K; *R*_f_ = 0.58 (hexane–ethyl acetate, 2:1 v/v)]. Single crystals suitable for X-ray diffraction were prepared by slow evaporation of a solution of the title compound in acetone at room temperature.

### Refinement

All H atoms were positioned geometrically and refined using a riding model, with C—H = 0.95 Å for aryl and 0.98 Å for methyl H atoms. *U*_iso_(H) = 1.2*U*_eq_(C) for aryl and 1.5*U*_eq_(C) for methyl H atoms. The positions of methyl hydrogens were optimized rotationally. The F1 atom of the 3-fluorophenyl ring is disordered over two positions with site occupancy factors, from refinement of 0.583 (5) (part A) and 0.417 (5) (part B). The distance of equivalent C-F pairs were restrained to 1.330 (5) Å using the SHELXL-97 command DFIX, and displacement ellipsoids of F1 set were restrained using the SHELXL-97 command ISOR.

### Crystal data

**Table:**

C_15_H_10_BrFO_2_S	*F*(000) = 704
*M**_r_* = 353.20	*D*_x_ = 1.681 Mg m^−^^3^
Monoclinic, *P*2_1_/*c*	Mo *K*α radiation, λ = 0.71073 Å
Hall symbol: -P 2ybc	Cell parameters from 3368 reflections
*a* = 13.0488 (4) Å	θ = 2.4–26.5°
*b* = 11.1874 (3) Å	µ = 3.10 mm^−^^1^
*c* = 9.9295 (3) Å	*T* = 173 K
β = 105.709 (2)°	Block, colourless
*V* = 1395.39 (7) Å^3^	0.25 × 0.24 × 0.13 mm
*Z* = 4	

### Data collection

**Table:**

Bruker SMART APEXII CCD diffractometer	3460 independent reflections
Radiation source: rotating anode	2392 reflections with *I* > 2σ(*I*)
Graphite multilayer monochromator	*R*_int_ = 0.048
Detector resolution: 10.0 pixels mm^-1^	θ_max_ = 28.3°, θ_min_ = 1.6°
φ and ω scans	*h* = −17→17
Absorption correction: multi-scan (*SADABS*; Bruker, 2009)	*k* = −13→14
*T*_min_ = 0.494, *T*_max_ = 0.746	*l* = −13→12
13225 measured reflections	

### Refinement

**Table:**

Refinement on *F*^2^	Primary atom site location: structure-invariant direct methods
Least-squares matrix: full	Secondary atom site location: difference Fourier map
*R*[*F*^2^ > 2σ(*F*^2^)] = 0.043	Hydrogen site location: difference Fourier map
*wR*(*F*^2^) = 0.115	H-atom parameters constrained
*S* = 1.03	*w* = 1/[σ^2^(*F*_o_^2^) + (0.0477*P*)^2^ + 0.9756*P*] where *P* = (*F*_o_^2^ + 2*F*_c_^2^)/3
3460 reflections	(Δ/σ)_max_ < 0.001
192 parameters	Δρ_max_ = 0.61 e Å^−^^3^
14 restraints	Δρ_min_ = −1.10 e Å^−^^3^

### Special details

**Table:**

Geometry. All esds (except the esd in the dihedral angle between two l.s. planes) are estimated using the full covariance matrix. The cell esds are taken into account individually in the estimation of esds in distances, angles and torsion angles; correlations between esds in cell parameters are only used when they are defined by crystal symmetry. An approximate (isotropic) treatment of cell esds is used for estimating esds involving l.s. planes.
Refinement. Refinement of F^2^ against ALL reflections. The weighted R-factor wR and goodness of fit S are based on F^2^, conventional R-factors R are based on F, with F set to zero for negative F^2^. The threshold expression of F^2^ > 2sigma(F^2^) is used only for calculating R-factors(gt) etc. and is not relevant to the choice of reflections for refinement. R-factors based on F^2^ are statistically about twice as large as those based on F, and R- factors based on ALL data will be even larger.

### Fractional atomic coordinates and isotropic or equivalent isotropic displacement parameters (Å^2^)

**Table:**

	*x*	*y*	*z*	*U*_iso_*/*U*_eq_	Occ. (<1)
Br1	0.78842 (3)	0.16727 (3)	0.52938 (5)	0.06445 (17)	
S1	0.70738 (6)	0.67272 (7)	0.23040 (7)	0.03887 (19)	
O1	0.93643 (16)	0.67342 (18)	0.5796 (2)	0.0384 (5)	
O2	0.7178 (2)	0.5873 (2)	0.1208 (2)	0.0595 (7)	
C1	0.8025 (2)	0.6387 (3)	0.3879 (3)	0.0333 (6)	
C2	0.8265 (2)	0.5272 (3)	0.4624 (3)	0.0326 (6)	
C3	0.7894 (2)	0.4090 (3)	0.4423 (3)	0.0385 (7)	
H3	0.7343	0.3862	0.3625	0.046*	
C4	0.8371 (3)	0.3279 (3)	0.5442 (4)	0.0426 (7)	
C5	0.9200 (3)	0.3574 (3)	0.6612 (3)	0.0445 (8)	
H5	0.9500	0.2978	0.7285	0.053*	
C6	0.9583 (2)	0.4726 (3)	0.6796 (3)	0.0411 (7)	
H6	1.0153	0.4945	0.7578	0.049*	
C7	0.9100 (2)	0.5543 (3)	0.5793 (3)	0.0345 (6)	
C8	0.8706 (2)	0.7221 (3)	0.4607 (3)	0.0352 (6)	
C9	0.8858 (3)	0.8504 (3)	0.4392 (4)	0.0463 (8)	
H9A	0.8629	0.8968	0.5096	0.069*	
H9B	0.8435	0.8738	0.3456	0.069*	
H9C	0.9613	0.8662	0.4482	0.069*	
C10	0.5915 (2)	0.6284 (3)	0.2817 (3)	0.0387 (7)	
C11	0.5255 (3)	0.5431 (3)	0.2057 (4)	0.0532 (9)	
H11	0.5429	0.5013	0.1317	0.064*	
C12	0.4339 (3)	0.5211 (4)	0.2412 (6)	0.0788 (15)	0.583 (5)
H12	0.3870	0.4631	0.1877	0.095*	0.583 (5)
C12'	0.4339 (3)	0.5211 (4)	0.2412 (6)	0.0788 (15)	0.42
F1'	0.3631 (4)	0.4412 (5)	0.1945 (7)	0.083 (2)	0.417 (5)
C13	0.4043 (3)	0.5740 (6)	0.3450 (6)	0.0877 (17)	
H13	0.3394	0.5553	0.3661	0.105*	
C14	0.4731 (3)	0.6561 (5)	0.4180 (4)	0.0805 (17)	0.583 (5)
F1	0.4478 (3)	0.6915 (4)	0.5278 (4)	0.0791 (15)	0.583 (5)
C14'	0.4731 (3)	0.6561 (5)	0.4180 (4)	0.0805 (17)	0.42
H14'	0.4556	0.6940	0.4944	0.097*	0.417 (5)
C15	0.5663 (3)	0.6885 (4)	0.3896 (3)	0.0542 (10)	
H15	0.6111	0.7490	0.4414	0.065*	

### Atomic displacement parameters (Å^2^)

**Table:**

	*U*^11^	*U*^22^	*U*^33^	*U*^12^	*U*^13^	*U*^23^
Br1	0.0542 (2)	0.0364 (2)	0.0981 (4)	0.00465 (16)	0.0128 (2)	0.00779 (18)
S1	0.0434 (4)	0.0439 (4)	0.0301 (4)	0.0032 (3)	0.0112 (3)	0.0067 (3)
O1	0.0351 (10)	0.0423 (12)	0.0376 (11)	−0.0004 (9)	0.0096 (8)	−0.0034 (9)
O2	0.0720 (16)	0.0729 (18)	0.0372 (12)	0.0076 (14)	0.0210 (11)	−0.0055 (11)
C1	0.0307 (13)	0.0405 (16)	0.0317 (14)	0.0033 (12)	0.0134 (11)	0.0013 (12)
C2	0.0280 (12)	0.0398 (16)	0.0324 (14)	0.0050 (12)	0.0125 (11)	0.0011 (12)
C3	0.0323 (13)	0.0404 (17)	0.0427 (16)	0.0037 (13)	0.0099 (12)	−0.0013 (13)
C4	0.0384 (15)	0.0360 (17)	0.0557 (19)	0.0062 (13)	0.0163 (14)	0.0028 (14)
C5	0.0433 (17)	0.0472 (19)	0.0441 (17)	0.0180 (14)	0.0139 (14)	0.0079 (14)
C6	0.0365 (15)	0.050 (2)	0.0343 (15)	0.0097 (14)	0.0059 (12)	−0.0037 (13)
C7	0.0313 (13)	0.0379 (16)	0.0361 (15)	0.0031 (12)	0.0123 (11)	−0.0020 (12)
C8	0.0339 (14)	0.0410 (17)	0.0353 (15)	0.0030 (13)	0.0170 (12)	0.0021 (13)
C9	0.0479 (18)	0.0395 (18)	0.055 (2)	−0.0038 (14)	0.0197 (15)	0.0019 (15)
C10	0.0347 (14)	0.0477 (18)	0.0300 (14)	0.0053 (13)	0.0025 (11)	0.0084 (13)
C11	0.0481 (18)	0.051 (2)	0.055 (2)	0.0010 (16)	0.0041 (16)	0.0024 (16)
C12	0.044 (2)	0.083 (3)	0.101 (4)	−0.009 (2)	0.006 (2)	0.025 (3)
C12'	0.044 (2)	0.083 (3)	0.101 (4)	−0.009 (2)	0.006 (2)	0.025 (3)
F1'	0.068 (3)	0.070 (4)	0.101 (4)	−0.032 (3)	0.007 (3)	−0.012 (3)
C13	0.0324 (18)	0.139 (5)	0.090 (3)	−0.001 (2)	0.014 (2)	0.040 (3)
C14	0.042 (2)	0.151 (5)	0.050 (2)	0.012 (3)	0.0150 (17)	0.012 (3)
F1	0.057 (2)	0.120 (4)	0.069 (3)	0.005 (2)	0.0304 (19)	−0.028 (2)
C14'	0.042 (2)	0.151 (5)	0.050 (2)	0.012 (3)	0.0150 (17)	0.012 (3)
C15	0.0370 (16)	0.086 (3)	0.0367 (17)	0.0038 (17)	0.0056 (13)	−0.0030 (17)

### Geometric parameters (Å, º)

**Table:**

Br1—C4	1.898 (3)	C6—H6	0.9500
Br1—O2^i^	3.200 (3)	C8—C9	1.472 (4)
S1—O2	1.482 (3)	C9—H9A	0.9800
S1—C1	1.755 (3)	C9—H9B	0.9800
S1—C10	1.792 (3)	C9—H9C	0.9800
O1—C8	1.371 (3)	C10—C11	1.367 (5)
O1—C7	1.377 (4)	C10—C15	1.378 (5)
C1—C8	1.355 (4)	C11—C12	1.357 (6)
C1—C2	1.441 (4)	C11—H11	0.9500
C2—C7	1.393 (4)	C12—C13	1.333 (7)
C2—C3	1.404 (4)	C12—H12	0.9500
C3—C4	1.376 (4)	C13—C14	1.349 (7)
C3—H3	0.9500	C13—H13	0.9500
C4—C5	1.396 (5)	C14—F1	1.284 (4)
C5—C6	1.377 (5)	C14—C15	1.370 (5)
C5—H5	0.9500	C15—H15	0.9500
C6—C7	1.373 (4)		
			
C4—Br1—O2^i^	159.51 (11)	C1—C8—C9	133.6 (3)
O2—S1—C1	109.35 (14)	O1—C8—C9	115.8 (3)
O2—S1—C10	106.41 (16)	C8—C9—H9A	109.5
C1—S1—C10	97.72 (13)	C8—C9—H9B	109.5
C8—O1—C7	106.5 (2)	H9A—C9—H9B	109.5
C8—C1—C2	107.6 (2)	C8—C9—H9C	109.5
C8—C1—S1	122.0 (2)	H9A—C9—H9C	109.5
C2—C1—S1	130.4 (2)	H9B—C9—H9C	109.5
C7—C2—C3	119.0 (3)	C11—C10—C15	121.5 (3)
C7—C2—C1	104.6 (3)	C11—C10—S1	119.4 (3)
C3—C2—C1	136.4 (3)	C15—C10—S1	118.9 (3)
C4—C3—C2	116.6 (3)	C12—C11—C10	116.8 (4)
C4—C3—H3	121.7	C12—C11—H11	121.6
C2—C3—H3	121.7	C10—C11—H11	121.6
C3—C4—C5	123.4 (3)	C13—C12—C11	125.4 (4)
C3—C4—Br1	119.5 (2)	C13—C12—H12	117.3
C5—C4—Br1	117.1 (2)	C11—C12—H12	117.3
C6—C5—C4	120.2 (3)	C12—C13—C14	115.3 (4)
C6—C5—H5	119.9	C12—C13—H13	122.3
C4—C5—H5	119.9	C14—C13—H13	122.3
C7—C6—C5	116.6 (3)	F1—C14—C13	112.3 (4)
C7—C6—H6	121.7	F1—C14—C15	122.6 (4)
C5—C6—H6	121.7	C13—C14—C15	124.6 (4)
C6—C7—O1	125.2 (3)	C14—C15—C10	116.3 (4)
C6—C7—C2	124.2 (3)	C14—C15—H15	121.8
O1—C7—C2	110.6 (2)	C10—C15—H15	121.8
C1—C8—O1	110.7 (3)		
			
O2—S1—C1—C8	127.1 (2)	C3—C2—C7—O1	178.8 (2)
C10—S1—C1—C8	−122.4 (3)	C1—C2—C7—O1	0.4 (3)
O2—S1—C1—C2	−51.6 (3)	C2—C1—C8—O1	−1.2 (3)
C10—S1—C1—C2	58.9 (3)	S1—C1—C8—O1	179.78 (19)
C8—C1—C2—C7	0.5 (3)	C2—C1—C8—C9	−179.9 (3)
S1—C1—C2—C7	179.4 (2)	S1—C1—C8—C9	1.2 (5)
C8—C1—C2—C3	−177.5 (3)	C7—O1—C8—C1	1.4 (3)
S1—C1—C2—C3	1.3 (5)	C7—O1—C8—C9	−179.7 (3)
C7—C2—C3—C4	2.1 (4)	O2—S1—C10—C11	−9.5 (3)
C1—C2—C3—C4	179.9 (3)	C1—S1—C10—C11	−122.3 (3)
C2—C3—C4—C5	−1.5 (5)	O2—S1—C10—C15	176.0 (3)
C2—C3—C4—Br1	177.6 (2)	C1—S1—C10—C15	63.1 (3)
O2^i^—Br1—C4—C3	−138.1 (3)	C15—C10—C11—C12	0.4 (5)
O2^i^—Br1—C4—C5	41.0 (5)	S1—C10—C11—C12	−174.0 (3)
C3—C4—C5—C6	0.0 (5)	C10—C11—C12—C13	−1.3 (7)
Br1—C4—C5—C6	−179.1 (2)	C11—C12—C13—C14	0.3 (7)
C4—C5—C6—C7	0.9 (5)	C12—C13—C14—F1	−170.9 (5)
C5—C6—C7—O1	179.6 (3)	C12—C13—C14—C15	1.7 (7)
C5—C6—C7—C2	−0.3 (5)	F1—C14—C15—C10	169.4 (4)
C8—O1—C7—C6	179.0 (3)	C13—C14—C15—C10	−2.5 (7)
C8—O1—C7—C2	−1.1 (3)	C11—C10—C15—C14	1.4 (5)
C3—C2—C7—C6	−1.3 (4)	S1—C10—C15—C14	175.8 (3)
C1—C2—C7—C6	−179.7 (3)		

Symmetry code: (i) *x*, −*y*+1/2, *z*+1/2.

### Hydrogen-bond geometry (Å, º)

**Table:**

*D*—H···*A*	*D*—H	H···*A*	*D*···*A*	*D*—H···*A*
C5—H5···O1^ii^	0.95	2.50	3.429 (4)	167
C9—H9*A*···O2^iii^	0.98	2.44	3.269 (4)	142

Symmetry codes: (ii) −*x*+2, *y*−1/2, −*z*+3/2; (iii) *x*, −*y*+3/2, *z*+1/2.

## References

[bb1] Brandenburg, K. (1998). *DIAMOND* Crystal Impact GbR, Bonn, Germany.

[bb2] Bruker (2009). *APEX2*, *SADABS* and *SAINT* Bruker AXS Inc., Madison, Wisconsin, USA.

[bb3] Choi, H. D., Seo, P. J. & Lee, U. (2012). *Acta Cryst.* E**68**, o1298. 10.1107/S160053681201389X PMC3344444 22590206

[bb4] Choi, H. D., Seo, P. J., Son, B. W. & Lee, U. (2010*a*). *Acta Cryst.* E**66**, o1297. 10.1107/S1600536810016181 PMC2979595 21579394

[bb5] Choi, H. D., Seo, P. J., Son, B. W. & Lee, U. (2010*b*). *Acta Cryst.* E**66**, o2721. 10.1107/S1600536810038870 PMC3009343 21588933

[bb6] Farrugia, L. J. (1997). *J. Appl. Cryst.* **30**, 565.

[bb7] Politzer, P., Lane, P., Concha, M. C., Ma, Y. & Murray, J. S. (2007). *J. Mol. Model.* **13**, 305–311. 10.1007/s00894-006-0154-7 17013631

[bb8] Sheldrick, G. M. (2008). *Acta Cryst.* A**64**, 112–122. 10.1107/S0108767307043930 18156677

